# Immunohistochemical assessment of galectin-3 during pre-implantation in mouse endometrium 

**Published:** 2013-02

**Authors:** Mahmoud Orazizadeh, Layasadat Khorsandi, Ghasem Saki

**Affiliations:** 1 *Cellular and Molecular Research Center (CMRC), Ahvaz Jundishapur University of Medical Sciences, Ahvaz, Iran.*; 2 *Department of Anatomical Sciences, Faculty of Medicine, Ahvaz Jundishapur University of Medical Sciences, Ahvaz, Iran.*

**Keywords:** *Endometrium*, *Estrus phase*, *Galectin-3*, *Preimplantation*

## Abstract

**Background: **Galectin-3 (Gal-3), a β-galactoside-binding lectin, is a multifunctional lectin that involves in a number of critical biological processes.

**Objective:** The purpose of this study was to investigate the expression pattern of Gal-3 in mouse endometrium during estrus phase of estrous cycle and pre-implantation.

**Materials and Methods:** In this experimental study 42 NMRI female mice were divided in seven different groups. Ovulation in NMRI female mice was stimulated by injecting hMG and hCG. Estrus phase was considered as stimulated and un-stimulated groups. The other groups of mice were mated, and the day of vaginal plug formation was considered as the day 1 of pregnancy. The mice of all groups were sacrificed on different days of pre-implantation period and their uterine horns were fixed and avidin- biotin complex method of immunohistochemistry (IHC) was applied.

**Results:** In estrus group, Gal-3 immunoreactivity in luminal epithelium was strong, in stromal cells very strong, in glandular epithelium very weak and endothelial cells very strong. No identifiable difference was observed in un-stimulated and stimulated estrus phase. In test groups, days 1-2, insignificant difference of Gal-3 expression was observed. On day 3, luminal epithelium and stromal cells showed significant decrease in comparison to estrus and day 1 (p=0.001). On the 4^th^ and 5^th^ days, luminal epithelium and stromal cells showed significant decrease in comparison to estrus phase and days 1-3 (p=0.0001).

**Conclusion:** The data suggested that successful implantation is probably associated with the downregulation of Gal-3 in the mouse endometrium at the beginning of pregnancy.

## Introduction

The human endometrium is a unique tissue because of its morphological and functional characteristics. The implantation process of blastocyst in the endometrium only takes place at a special time which is called "the window of implantation" (1). 

During this postovulatory phase, under the influence of progesterone, glandular secretion and stromal decidualization, and also changes in the immune cells distribution, become the dominant features of the endometrium (2-3). In spite of extensive researches, the details of uterine changes during critical phenomena such as pregnancy, preimplantation and implantation, are still not understood properly. 

A number of critical functions have been described so far for lectins family. Lectins are unique group of proteins that presented an important field for the biological researches in recent dacade. A very interesting and noticeable family between them is galectins (4). 

Most galectins, are di- or multivalent and are therefore able to act as versatile cross-linkers of glycosylated cell surface molecules (5). Linkage of these proteins to a variety of important processes, including neoplastic transformation and tumor invasiveness, cell adhesion, cellular proliferation, cell-cell, and cell-matrix interactions and local immunomodulation, has generally been attributed to their ability to recognize specific glycoconjugates by means of their binding sites (6). 

Galectins have been shown to either promote or suppress some critical processes, depending on the galectin member studied as well as experimental conditions (7). Galectin-3 (Gal-3), a member of galectins family, is a multifunctional protein which is implicated in various biological processes including cell proliferation, apoptosis, angiogenesis, tumor progression and metastasis (8). This specific member of galectins family, possesses bivalent capacity because of its β-galactoside-binding domain and carbohydrate recognition domain (CRD).This characteristic causes a vast capability in linking with a variety of biological molecules and thereby involving in intermolecular interactions (4, 8).

Several studies have so far focused on the role of galectin-3 in uterus receptivity in various conditions such as menstrual cycle and implantation (9, 10). Wolff *et al* have demonstrated that Gal-3 expression in human endometrium is a cycle dependent manner (9). Very recently Yang *et al* have reported that Gal-3 expression is increased during embryonic implantation by using in vitro and in vivo techniques (10). 

The results of the recent work in comparison to the other in vitro and in vivo studies presented controversial findings. Therefore, this study was aimed to do a day-by-day and in vivo assessment of pre-implantation to show more details related to implantation. Alterations of pre-implantation period and its characteristics could present an insight related to normal and abnormal implantation. The present study was undertaken to investigate the expression pattern of Gal-3 in mouse endometrium during estrus phase of estrous cycle and pre-implantation period. 

## Materials and methods

**Animals**

In this experimental study, all experiments were carried out according to the criteria required by Ethical Committee of Ahvaz Jundishapur University of Medical Sciences. Forty two NMRI female mice were divided into seven groups. The mice were purchased from Animal house of Ahvaz Jundishapur University of Medical Sciences. Animals were housed in a room under a controlled light/dark cycle and temperature and fed with a standard laboratory program. Two groups were considered for estrus phase under stimulated and unstimulated conditions.

Ovulation in NMRI female mice was stimulated by injecting hMG and hCG (10 IU each). The rest of mice were divided into five groups for the first to 5^th^ day of pregnancy. These 5 groups were mated, and the day of vaginal plug formation was considered as the first day of pregnancy. The mice of all groups were sacrificed on different days and their uterine horns were fixed in 10% formalin.

**Immunohistochemistry**

Immunohistochemical (IHC) study was applied as previously described using avidin- biotin complex (ABC) method (11, 12). Briefly, formalin fixed and paraffin embedded sections of mice uterine were deparaffinized in xylol and hydrated. Endogenous peroxidase activity was blocked by incubation in methanol containing 0.3% H_2_O_2_ for 15 min at room temperature. Antigen retrieval was applied by using citrate buffer (pH=6) for 15 min at 98^o^C. Then the sections incubated with mouse monoclonal primary anti-Gal-3 (B-9) (Santacruz, sc: 7480) at 1:50 diluted in phosphate buffered saline (PBS, pH=7.4) containing 10% normal goat serum (NGS). 

The secondary antibody (Biotinylated anti-mouse IgG) (ABC Peroxidase Mouse IgG staining Kit: Santacruz, sc-2017) was applied at 1:100 for 50 min. Negative control was used by omitting the primary antibody to assess the level of antigen-independent binding. Then, the sections were incubated with peroxidase-conjugated specimens for 30 min and diaminobenzidine (DAB) as chromogen and counterstained with hematoxylin. 

Three immunohistochemical sections from each animal were blindly assessed by two expert people. The staining intensity was estimated using a semiquatitative score, H-score, as previously described (12). The H-score was calculated for each section by application of the following algorithm: HSCORE=ΣP (I+1), where I is the intensity of staining (0-no staining, 1 for weak, 2 for moderate, and 3 for strong) and Pi is the percentage of stained cells for each intensity (0-100%) (12, 13). 

**Statistical analysis**

The data were analyzed using one-way ANOVA and followed by Mann-Whitney U test. All numerical data are represented as the Mean±Standard Deviation (Mean±SD). Version 15 of SPSS was used for all statistical analyses. The difference was considered statistically significant when p value was less than 0.05 (P<0.05).

## Results

**Estrus groups**

**Figure 1:**
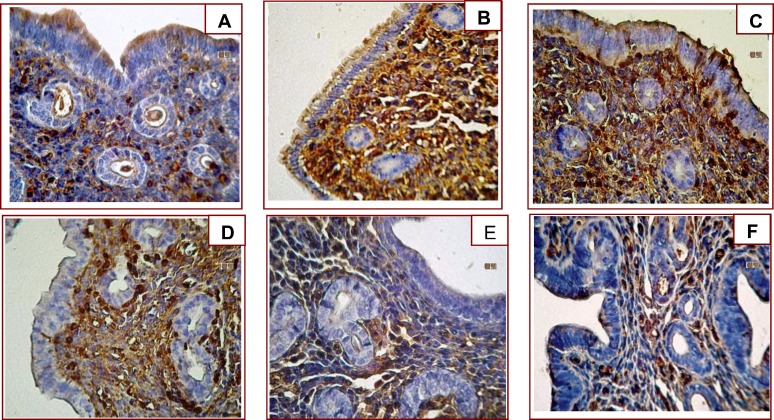
Immunoreactivity of galectin-3 in mouse endometrium of stimulated estrus phase (A), unstimulated estrus group (B), and during the 2^nd^ day of pregnancy (C), the 3^rd^ day of pregnancy (D), the 4^th^ day of pregnancy (E), and the 5^th^ day of pregnancy (F). X400

**Figure 2:**
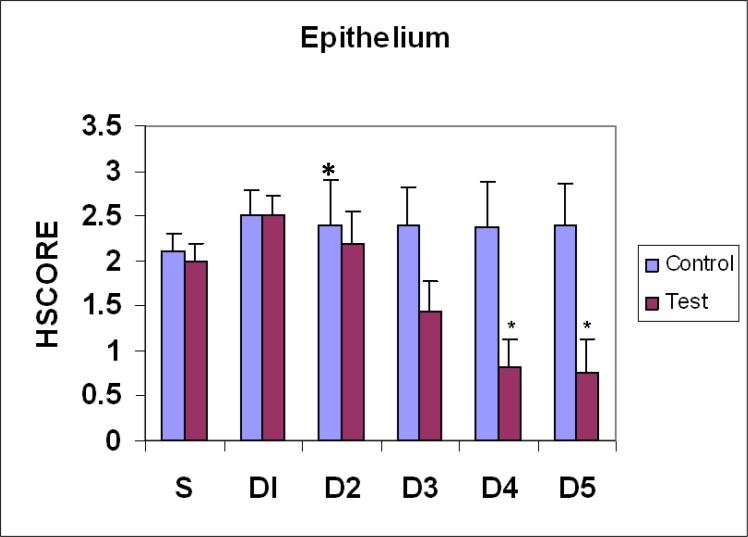
Comparison of galectin-3 expression pattern in epithelial cells of mouse endometrium during estrus phase (S) and different days (D1-D5) of pregnancy. (Mean±SEM), *, p<0.05

The pattern of Gal-3 expression was evaluated in various control and test groups. To assess the results properly, two un-stimulated and stimulated estrus groups were considered. Gal-3 immunoreactivity was strong in epithelial luminal cells, very strong in stromal and endothelial cells, and very weak in glandular epithelial cells. (Figure 1-A, B). No identifiable difference was observed in un-stimulated and stimulated estrus phase (Figure 2).

**Day 1-5 of pregnancy**

**Figure 3:**
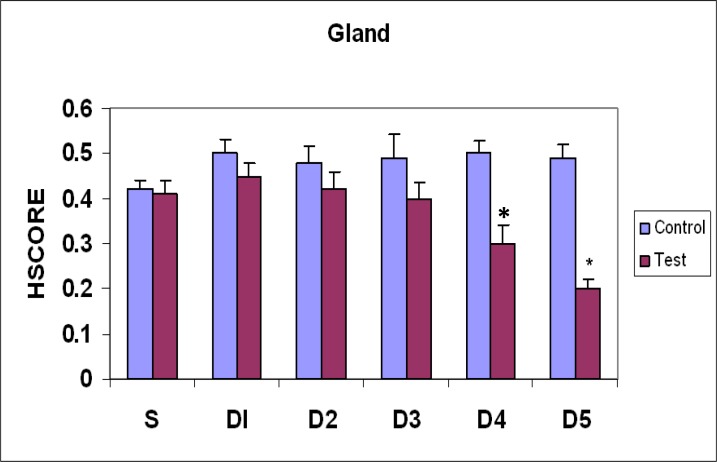
Comparison of galectin-3 expression pattern in glandular cells of mouse endometrium during estrus phase(S) and different days (D1-D5) of pregnancy. (Mean±SEM), *, p<0.05

**Figure4:**
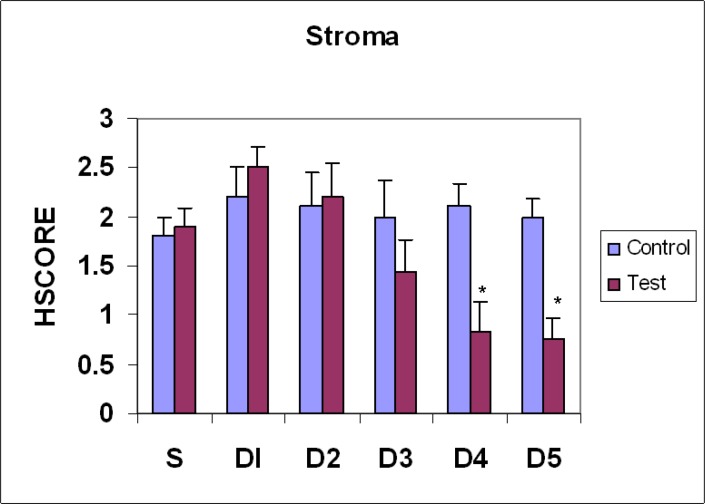
Comparison of galectin-3 expression pattern in stromal cells of mouse endometrium during estrus phase (S) and different days (D1-D5) of pregnancy. (Mean±SEM), *, p<0.05

On the 1^st^ day of pregnancy in the test group, non-significant difference of Gal-3 expression in all different cell types was observed compared to day 1 of control and estrus phase (Figure 2). On the 2^nd^ day of the test group, compared to estrus, control and day1 , no significant difference was observed (p>0.05) (Figures 1-C, 2-5). On the 3^rd^ day, epithelial luminal and stromal cells showed significant decrease in comparison with estrus and day 1 (p=0.001) (Figures 1-D, 2, 3). The modulation of epithelial luminal cells immunoreactivity was noticeable (Figure 2). No significant difference was observed in expression pattern of the other cell types (Figure 1-D) compared with the 2^nd^ day. 

On the 4^th^ day, epithelial luminal cells showed significant decrease in comparison with estrus and days 1and 2 (p=0.0001), and insignificant with day 3 (Figures 1-E, 2). In glandular epithelial cells, significant decrease was observed in comparison with control groups and day 1 of pregnancy (Figure 3). Stromal cells showed significant decrease in comparison to estrus and days 1-3 (p=0.0001) (Figure 4).Endothelial cells showed the same pattern of expression in control and test groups (not shown). On the 5^th^ day, epithelial luminal cells showed significant decrease in comparison with estrus and days 1-3 (p=0.0001), and insignificant with day 4 (Figures 1-F, 2). Glandular epithelial and stromal cells showed significant difference in comparison with control and days 1-3 (p=0.0001), and insignificant with the 4^th^ day (Figures 3, 4). No noticeable difference was observed in endothelial cells (not shown).

## Discussion

The present study showed the day-by-day alterations of the Gal-3 expression pattern in the various cell types of mouse uterus during estrus phase and pre-implantation under invivo condition. A differential expression pattern of Gal-3 was observed in different days of pre-implantation and estrus phase in various cell types including luminal epithelium, stromal and glandular epithelium cells. 

Regarding quite different functions of Gal-3 in various processes such as apoptosis, cell adhesion, and also its involvement in many signaling pathways in normal and abnormal cells, the present findings may open a new window in the functions of Gal-3 and provide more details of pre-implantation modulations. 

A number of studies have previously concentrated on the role of Gal-3 in various stages of implantation and pregnancy. For instance, In 1994, van den Brule *et al* showed that Gal-3 expression in human endometrium is decreased during first trimester of pregnancy. They also reported its abundance inversely correlated with trophoblast invasiveness during the course of gestation (14). 

The general idea in the present work is in agreement with the findings of van den Brule group. Both studies confirm a decreasing process of Gal-3 expression in a period that is linked to pre-implantation and implantation periods in human and mouse. Philips *et al* have reported that Gal-3 is expressed in uterine epithelia of pregnant mice immediately after implantation, but not in non-pregnant animals, or during the pre-implantation stages of pregnancy. They concluded that the function of Gal-3 is related specifically to pregnancy (15). 

The results of Philips *et al* study are in consistent with the current study. Taken together, the data could prepare a clear plan of alterations in endometrium during pre-implantation and after implantation that could be applied for detection of some kinds of disorders. Lee *et al* have studied the expression of Gal-3 in the murine utero-placental complex. They reported that Gal-3 is expressed in various cell types including Luminal epithelium and decidual cells of endometrium, and also natural killer cells, and trophoblast cells of placenta. They also reported that Gal-3 passes through extracellular matrix and resides in various compartments of endometrium (16). 

They have just focused on utero-placental complex, whereas in our study we concentrated on endometrium tissue as a critical target in pre-implantation stage. The rate of alterations in endometrium for window of implantation begins from the date of plug formation and undergoes a series of dynamic changes. Thus the expression, the absence or every change in the presentation of a molecule is a variable process and is related to a number of factors such as its role in the other biological phenomena. 

For example Gal-3 could participate in immune regulation and cell adhesion processes, and thus the behavior of this molecule could be explained by these characteristics. Studies have focused on the functions of galectins in modulation of immune system (21-22). Suitable function of endometrium or a successful implantation depends on the migration of leukocytes and aggregation of immune cells around the implantation site. galectins can play a critical role in regulation of migration and localization of leukocytes (16). 

Gal-3 has a role in regulation of immune response and inflammation and acts as a strong pro-inflammatory signal (21-22). Thus, its significant down regulation during pre-implantation leads to progress of implantation without critical immunologic and inflammatory side-effects. It seems that weak expression of Gal-3 is a critical process which promote a balanced immune reaction and a perfect implantation. In other words, Gal-3 downregulation suggests that this member of lectins family specifically involves in cell homeostasis to regulate endometrial functions, as previously explained (18). 

Vicovac *et al* have reported that Gal-3 expression in human embryo and maternal tissue are independent. They also reported that the expression of Gal-3 in uterine decidua is not related to embryo presence in uterine and utero-placental complex formation (19). 

Unlike Vicovac *et al* findings, our data shows that, the expression of Gal-3 is absolutely related to embryo presence in uterus and the progress of utero-placental complex formation is dependent on Gal-3 down regulation. The contradiction between two works may attribute to variations in methodology of study and the changes in applied biological systems. 

Although, Wolff *et al* in consistent with the present work, have shown that Gal-3 expression in human endometrium is a cycle dependent manner and during late secretory phase of the menstrual phase is increased significantly (9). In addition, we have recently showed that the Gal-3 expression in mouse testicular tissue is a stage dependent manner (20). Yang *et al* have lately reported that an increased Gal-3 expression after pregnancy is required for implantation of mice embryo (10). In their work, different invivo and invitro methods have been applied and they focused on in vitro results. The results of Real-time PCR, Western blot and immunohistochemistry are not properly matched and the days of up and down regulation in each method is different from another. 

In our study, we just used an invivo stimulated model for evaluation of Gal-3 expression pattern and assessed every day before implantation. Therefore, it is possible that the present results are not exactly the same as Yang *et al* results. Having multiple binding domains is a unique structural characteristic of the Gal-3 that enables it to associate with different other molecules directly or indirectly (23). In this regard, considering a variety of molecules with this capability are quite important. 

For instance, integrins and CD biomarkers are two important partners for galectins family that could effectively modified their functions. For example in a recent work, the localization between αvβ_3_ integrin and Gal-3 in human endometrium has been shown (23). In addition, this combination of Integrin-Gal-3 could associate indirectly through a heterodimeric transmembrane glycoprotein CD98 (24). 

CD98 is a 125-kDa glycoprotein that galectin-3 act as an endogenous cross-linker of this antigen, which result in the activation of integrin-mediated adhesion (25). The functions of these molecular interactions are properly associated and therefore more researches in this area are needed. In summary, very weak Gal-3 expression immediately before implantation is related to its critical role in regulation and balance of implantation in a mouse. 

The presence of Gal-3 in epithelial luminal and stromal cells, is possibly related to its involvement in regulating of implantation events such as modulation of immune reaction and cell adhesion. Therefore, further researches on Gal-3 partners and also other galectins is suggested to achieve more progress in this field.
